# Mild Water-Filtered Infrared-A Whole-Body Hyperthermia Reduces Pain in Patients with Fibromyalgia Syndrome—A Randomized Sham-Controlled Trial

**DOI:** 10.3390/jcm12082945

**Published:** 2023-04-18

**Authors:** Jost Langhorst, Anna K. Koch, Christina Kehm, Özlem Öznur, Harald Engler, Winfried Häuser

**Affiliations:** 1 Department of Internal and Integrative Medicine, Sozialstiftung Bamberg, 96049 Bamberg, Germany; 2 Department of Integrative Medicine, Medicinal Faculty, University of Duisburg-Essen, 96049 Bamberg, Germany; 3 Department of Pediatrics, Division of Oncology and Hematology, Charité Universitätsmedizin Berlin, Corporate Member of Freie Universität Berlin, Humboldt-Universität zu Berlin and Berlin Institute of Health, 13353 Berlin, Germany; 4 Institute of Medical Psychology and Behavioral Immunobiology, Center for Translational and Behavioral Neurosciences, University Hospital Essen, University of Duisburg-Essen, 45147 Essen, Germany; 5 Department of Psychosomatic Medicine and Psychotherapy, Technical University Munich, 81675 Munich, Germany

**Keywords:** fibromyalgia, whole-body hyperthermia, randomized controlled trial, integrative medicine

## Abstract

The challenging treatment situation of patients with fibromyalgia syndrome (FMS) requires additional therapy options. The effects of water-filtered infrared-A whole-body hyperthermia (WBH) versus sham hyperthermia on pain intensity were investigated in an outpatient setting within a two-armed randomized sham-controlled trial. *n* = 41 participants aged between 18 and 70 years with a medically confirmed diagnosis of FMS were randomized to WBH (intervention; *n* = 21) or sham hyperthermia (control; *n* = 20). Six treatments with mild water-filtered infrared-A WBH over a period of three weeks with at least one day in between treatments were applied. On average, the maximum temperature was 38.7 °C for a duration of approximately 15 min. The control group received exactly the same treatment except that an insulating foil between the patient and the hyperthermia device blocked most of the radiation. Primary outcome was pain intensity measured by the Brief Pain Inventory at week 4. Secondary outcomes included blood cytokine levels and FMS-related core symptoms and quality of life. Pain intensity at week 4 was significantly different between the groups in favor of WBH (*p* = 0.015). A statistically significant pain reduction in favor of WBH was also found at week 30 (*p* = 0.002). Mild water-filtered infrared-A WBH effectively reduced pain intensity at the end of treatment and follow-up.

## 1. Introduction

Fibromyalgia syndrome (FMS) is a chronic disorder characterized by chronic widespread pain, physical and/or mental fatigue, and nonrestorative sleep as core symptoms. It is often accompanied by various somatic and psychological symptoms such as headache, bowel problems, morning stiffness, anxiety, and depression. The worldwide prevalence varies between 0.2 and 6.6% [1]. Women with advancing age have a higher risk of the disease [2]. The pathophysiology of FMS remains unclear, but may involve altered central pain processing (central sensitization) [3], alterations in central nervous neurotransmitters [4], dysfunction of the sympathetic nervous system [5], small fiber pathology [6,7], and abnormality of microcirculation [8].

The treatment situation of FMS is often perceived as insufficient [9,10,11,12]. The most effective therapies are according to several evidence-based guidelines aerobic exercise, cognitive behavioral therapy (CBT), multimodal therapy [13], and some antidepressants (e.g., amitriptyline, duloxetine) [14]. Complementary treatments are also frequently requested by patients with FMS [15]. Heat applications were explicitly recommended as self-management strategies in the current S3-guidelines [16]. Mild water-filtered infrared-A (wIRA) whole-body hyperthermia (WBH) showed first promising results in FMS [17,18,19,20,21,22]. However, two of the cited studies were non-controlled [20,21] and three of the controlled trials were not randomized [18,19,22], which makes a bias on the results possible.

WBH works by increasing body-core temperature to create an artificial fever-like state [23]. Adverse effects were mostly physiological reactions to body heating and were short-lived. Besides an increased perfusion of tissues and organs and an acceleration of biochemical metabolic processes, data indicate immunological processes [24,25]. Thermal and non-thermal effects of wIRA-WBH act on cells, cellular structures, and substances, and possibly on nociceptors, and influence a variety of processes. Several studies indicate that hyperthermia can affect the autonomic nervous system, which in turn is linked with pain processing and control of immunological processes [26,27,28,29]. Tarner et al. [30] detected a reduction of proinflammatory cytokines after the application of hyperthermia in patients with ankylosing spondylitis. In rheumatic diseases, results at the molecular level explain the clinically demonstrable reduction in pain and the consequent reduced need for analgesics [31]. Further studies are required, to identify the pain-ameliorating (analgesic) mediators activated by the immune system during WBH. Especially changes in tumor necrosis factor (TNF)-α and interleukin (IL-)-6, IL-8, IL-10 should be monitored, as these cytokines are commonly associated with FMS [32,33,34,35,36].

The main objective of this randomized sham-controlled trial was to investigate the effect of mild wIRA-WBH on pain intensity in patients with FMS in an outpatient setting.

## 2. Materials and Methods

### 2.1. Design and Procedure

This prospective, monocentric, randomized, sham-controlled, single-blinded, 2-armed, parallel group trial was conducted from November 2020 to December 2021 at the Sozialstiftung Bamberg, Bamberg, Germany. The study was approved by the Ethics Committee of the Bayerische Landesärztekammer (BLÄK, approval number 20079), registered on clinicaltrials.gov (https://clinicaltrials.gov/ct2/show/NCT05135936 (accessed on 13 February 2023), and performed according to the declaration of Helsinki applying good clinical practice standards.

After signing all information consent forms and being medically assessed as eligible, participants were randomized into two groups. At the end of the study period participants were informed in written form about their group assignment.

### 2.2. Participants

Participants were recruited in cooperation with three German Fibromyalgia Associations, the German Rheumatism League, via public advertisements in local newspapers, the website of the hospital and information events at the clinic.

Patients (m, f) aged between 18 and 70 years with a medically confirmed diagnosis of FMS additionally reviewed by a physician according to ACR 2016 criteria during the screening visit and an average pain intensity of ≥4.0 were eligible. The latter was recorded via a pain diary, measuring the pain level four times a day 14 days prior to the eligibility assessment. Main exclusion criteria were severe somatic and psychiatric comorbidities, other chronic pain syndromes, the intake of opioids, cannabis, and immunosuppressive drugs, contraindications for hyperthermia such as body temperature >37.5 °C, heart failure, previous experience with WBH, or the participation in other clinical intervention studies (see Table S1).

### 2.3. Randomization

After being assessed as eligible by the study doctor, patients were randomly assigned to either the intervention or the control group by the study coordinator (SC). For that purpose, the study coordinator prepared opaque sealed envelopes containing either “A” (for intervention) or “B” (for control) in a 1:1 ratio with a block size of 10. The SC asked the patients to select one envelope, open it and to say out loud the containing letter, which was then protocolled. The meaning of the letters and group allocation was blinded to the patients, study doctor, and those being involved in the assessment of outcomes (questionnaires, blood samples) but not to the SC and therapists.

### 2.4. Intervention Group

Participants assigned to the intervention group received a total of six sessions with mild wIRA-WBH of 60 min over a period of three weeks with at least one day between each intervention session.

**Figure 1:**
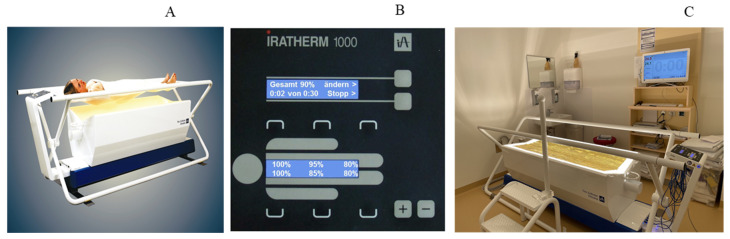
IRATHERM1000 hyperthermia device and sham condition. IRATHERM1000 hyperthermia device (**A**), control panel (**B**), hyperthermia device with attached insulating foil to create sham condition; the foil is able to reflect most of the radiation and avoid overheating (**C**).

The IRATHERM1000 hyperthermia device was used for this purpose (Von Ardenne Institut für Angewandte Medizinische Forschung GmbH, Dresden, Germany; Figure 1A).

The device stands out for its open design and lying comfort. It has six halogen radiators arranged longitudinal with three on each side including water. The resulting spectrum is called water-filtered infrared-A (wIRA), with typical absorption lines of water and free of infrared B and C. For wIRA, the relationship between irradiation of the skin and deeper lesions is much more favorable compared to conventional technical IR-devices. Maximum irradiance is 1400 W/m^2^ (corresponding to 100%) and can be adjusted in 5% intervals by a control panel (Figure 1B) for each radiator separately. According to the guideline for mild WBH (DGHT, 2018), the target body-core temperature for each session in the present study was 38.5 °C as measured rectally (Bluepoint Medical, accuracy of ±0.1 °C in the range of 25 °C to 50 °C). Irradiance was set on 80% (1120 W/m^2^) during the heating-up phase. When reaching the target temperature, irradiance was decreased to 40% (560 W/m^2^) to maintain body-core temperature until the end of the 60-min treatment period (plateau phase). This led to a further small increase in temperature in most of the subjects. Deviations from the planned irradiance were made according to the needs of the participants. While being treated subjects laid undressed on the device being covered by a white sheet and a reflection foil.

Rectal and axillar temperature, pulse and oxygen saturation (SpO_2_) were recorded continuously during the entire session and could be viewed at any time by the therapist, but not by the participant. After treatment, participants rested for about 30 min (resting-phase). Thus, one complete intervention was approximately 1.5 to 2 h. Subjects were permanently supervised and a physician was always on call. A discontinuation criterion of the treatment was a body-core temperature of more than 40 °C.

### 2.5. Control Group

For the control group, the treatment was framed in the patient information as “gentle hyperthermia”. The key difference between the two conditions was that subjects received significantly less heat. In order to achieve this, an insulating foil was attached on the IRATHERM1000 hyperthermia device (Figure 1C) able to reflect most of the infrared before reaching the body. For this purpose, the irradiance was set to 80% for about 30 min and reduced to 40% afterwards. On average, with the reflective foil in place, 2.2 +/− 0.4% of the usual radiation was measured in the radiation area at various points at patient level (in the head and pelvic area, each centered and 15 cm from the edge). These were carried out with calibrated radiometers of the type ILT400 and ILT2400 from the company International Light Technologies Inc., Peabody, MA 01960, USA. The conduct did not otherwise differ from that of the intervention group.

### 2.6. Outcomes

Demographic characteristics were recorded at baseline (week 0). Clinical characteristics were captured at baseline (week 0), one week after the end of treatment (week 4/postintervention), and two and six months after the end of the treatment (week 12/2 months follow-up; week 30/6 months follow-up).

#### 2.6.1. Primary Outcome

The primary outcome was pain intensity measured by the Brief Pain Inventory (BPI) [37,38] at week 4. The subscale describes a mean score ranging from 1–10 covering the “strongest”, “lowest”, and “average” pain of the past 24 h and the current pain. Higher scores indicate higher average pain intensity. A pain reduction of 30% or more is considered to be clinically relevant [39].

#### 2.6.2. Secondary Outcomes

Pain impairment was measured by the BPI [37,38]. FMS-related quality of life was measured using the 19-item Fibromyalgia Impact Questionnaire (FIQ) [40,41]. Depression was assessed by the Patient Health Questionnaire-9 (PHQ-9) [42]. Fatigue was assessed by the 20-item Multidimensional Fatigue Inventory (MFI-20) [43,44]. Sleep quality was assessed by the 19-item Pittsburgh Sleep Quality Index (PSQI) [45,46]. General health-related quality of life was assessed with the validated 36-item Short Form Health Survey (SF-36) [47,48]. Anxiety was assessed with the Generalized Anxiety Disorder Scale (GAD-7) [49]. Due to organizational problems anxiety was only measured in a subsample of *n* = 28 patients (WBH: *n* = 15; sham: *n* = 13). Sensory and affective pain were measured using a subscale of the Short-Form McGill Pain Questionnaire (SF-MPQ) [50]. Somatic symptom load was assessed by the Patient Health Questionnaire-15 (PHQ-15) [51]. It contains 13 items covering various physical complaints and two items of the depression module (PHQ-9) [42]. All questionnaires were used in the German version. For detailed description see Table S2.

#### 2.6.3. Blood Parameters

Blood samples were collected at baseline (T0), immediately after the last treatment session (T1) and at the following week (week 4, T2) to determine systemic concentrations of key pro- and anti-inflammatory cytokines (i.e., TNF-α, IL-6, IL-8, and IL-10). Average daily interval of T1 and T2 blood collection was 4.16 ± 1.08 days. At these timepoints, also a routine laboratory testing with C-reactive protein (CRP), erythrocyte cell sedimentation rate (ESR) and white blood cell (WBC) differential was performed. Venous blood was collected in tubes containing EDTA (S-Monovette, Sarstedt, Nümbrecht, Germany) and plasma was separated by centrifugation (2000× *g*, 10 min, 4 °C) and stored at −80 °C until analyses. Plasma cytokine levels were determined by high-sensitive enzyme-linked immunosorbent assays (ELISA) with detection limits of 0.022 pg/mL for TNF-α, 0.031 pg/mL for IL-6, 0.130 pg/mL for IL-8, and 0.090 pg/mL for IL-10. Serum concentrations of CRP, ESR and WBC differential were analyzed by the local clinical laboratory.

#### 2.6.4. Safety

Safety was assessed by spontaneous patient reports of side effects during treatment sessions and by review of the pain diary, which included an open-end field for comments on daily basis. Patients were instructed before every treatment session to report on side effects.

### 2.7. Statistical Analysis

Intention-to-treat analyses were performed. Missing values were imputed 50 times using the Markov–Chain–Montecarlo-procedure. The primary outcome was evaluated using an analysis of covariance (ANCOVA) with baseline scores as covariate to control for group differences in pain intensity at baseline and with group as between-subject factor. Blood parameters were evaluated using multivariate repeated-measures analysis of variance (rmANOVA). Post hoc *t*-tests were calculated if rmANOVA revealed significant interaction effects. Group differences were considered as statistically significant if the two-sided *p*-value was <0.05. Secondary parameters were compared exploratively using ANCOVA. Due to the exploratory nature regarding secondary outcomes, no adjustments for multiple comparisons were made. Partial eta-squared (η^2^_p_) was reported as an effect-size estimator. A partial eta square of 0.01, 0.06, and 0.14 corresponds to a small, medium, and large effect, respectively. All results are reported as mean ± standard deviation (M ± SD). Analyses were performed using the Statistical Package for Social Sciences software (IBM SPSS Statistics for Windows, release 28.0; IBM Corporation, Armonk, NY, USA).

### 2.8. Sample Size

A moderate effect size (Cohen’s *d* = 0.60) with clinical relevance was assumed based on prior results by Brockow et al. [17]. Using G-Power with a two-sided significance level of 5% and a power of 1 − β = 80% a sample size of 28 subjects was calculated. In order to compensate for a possible loss rate of 30%, approx. 20 subjects per group were planned to be enrolled in the study.

## 3. Results

### 3.1. Participants

Pre-screenings via telephone were made with 79 people, of which 44 met the main inclusion criteria (56%) and were medically assessed for eligibility at the clinic. Finally, 41 subjects (93%) fulfilled inclusion and exclusion criteria and were randomized (WBH, *n* = 21, sham, *n* = 20) and analyzed (see Figure S1). Demographic and clinical characteristics of the sample are described in Table 1.

### 3.2. Primary Outcome

**Figure 2:**
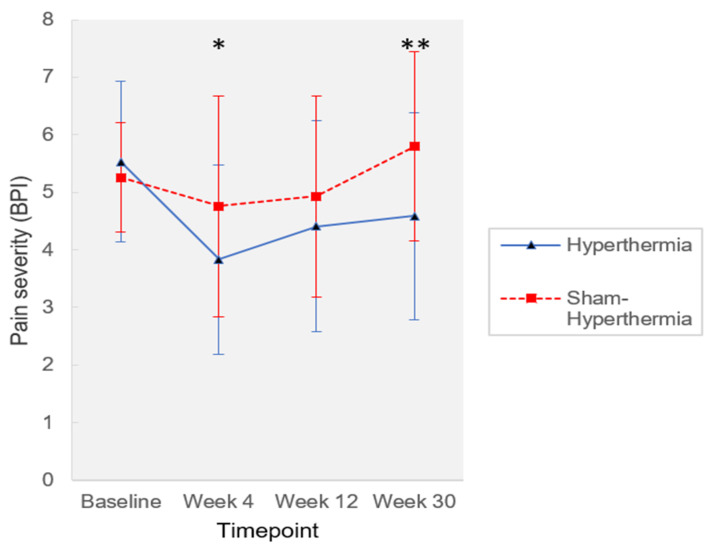
Pain intensity during the study period. Pain intensity during the study period as measured by the Brief Pain Inventory (BPI; 0–10; mean ± standard deviation); hyperthermia *n* = 21, sham hyperthermia *n* = 20; * *p* < 0.05; ** p < 0.01.

Pain intensity at week 4 was statistically significantly different between the groups in favor of mild WBH (*p* = 0.015, *η^2^*_p_ = 0.146, Figure 2).

The intervention group showed an average reduction in pain intensity from baseline of 1.7 points (0–10; −30.7%; T0: 5.53 ± 1.40, T1: 3.83 ± 1.64), while pain intensity in the control group was reduced by 0.5 points (−9.5%; T0: 5.26 ± 0.95, T1: 4.76 ± 1.92). In addition, a clinically relevant reduction in pain of 30% from baseline was found in 10 patients of the intervention group and in 4 patients of the control group (Chi^2^ = 2.4, *p* = 0.13).

### 3.3. Secondary Outcomes

A statistically significant pain reduction in favor of the intervention group was found at week 30 (*p* = 0.002, *η^2^*_p_ = 0.233) with an average reduction of pain intensity of 0.9 points (0–10; −17.0%; T0: 5.53 ± 1.40, T3: 4.59 ± 1.80). Pain intensity in the control group was increased by 0.5 points (+10.3%; T0: 5.26 ± 0.95, T3: 5.80 ± 1.65). Pain impairment measured at week 30 was statistically significantly different between the groups in favor of mild WBH (*p* = 0.008, *η^2^*_p_ = 0.172; mild WBH, T3: 3.96 ± 2.45; sham, T3: 5.13 ± 1.77). Mental health index score as measured with the SF-36 at week 4 showed statistically significant differences between the groups in favor of mild WBH (*p* = 0.043, *η^2^*_p_ = 0.103; mild WBH, T0: 38.77 ± 11.60, T1: 49.50 ± 9.47; sham, T0: 43.88 ± 9.72, T1: 47.12 ± 12.35). Secondary outcomes are presented in Table S3. The explorative nature of secondary analyses has to be considered when interpreting *p*-values.

### 3.4. Immunological Changes

Analyses including all blood parameters as outcome variables revealed a statistically significant time × group interaction for lymphocyte (*p* = 0.009, *η^2^*_p_ = 0.115) and neutrophil counts (*p* = 0.045, *η^2^*_p_ = 0.077).

**Figure 3:**
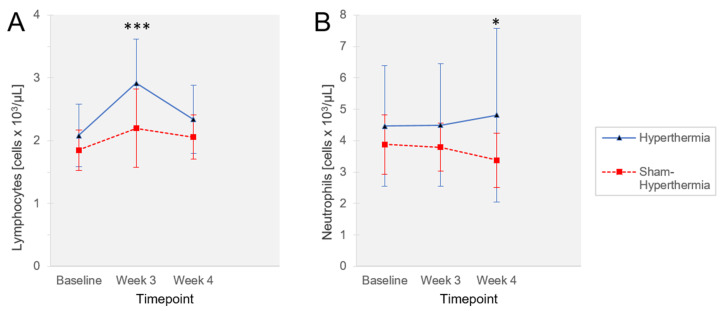
Lymphocytes and neutrophils during the study period. (**A**) Lymphocytes (cells × 10^3^/µL) and (**B**) neutrophils (cells × 10^3^/µL) in venous blood at intervention start (T0, baseline), intervention end (T1, Week 3) and after several days after final session (T2, Week 4) (mean ± standard deviation). Hyperthermia *n* = 21, sham hyperthermia *n* = 20. Post hoc *t*-test (hyperthermia vs. sham); *** *p* ≤ 0.001; * p = 0.05.

Post hoc *t*-tests showed for lymphocytes a statistically significant group difference (*p* ≤ 0.001) at T1 (mild WBH: 2.91 ± 0.71, sham: 2.20 ± 0.62; cells × 10^3^/µL) with a greater increase in lymphocyte count by mild WBH (Figure 3A). Neutrophil numbers revealed a statistically significant group difference (*p* = 0.033) at T2 (mild WBH: 4.82 ± 2.77, sham: 3.38 ± 0.86; cells × 10^3^/µL) with higher scores in the mild WBH group (Figure 3B). Multilevel analyses revealed negative effects of lymphocytes on pain intensity, stating that an increase in lymphocyte count results in a statistically significant reduction in pain, in the timeframe T0 to T1 with *p* ≤ 0.001 (B = −0.905 ± 0.257) and in the timeframe T0 to T2 with *p* = 0.017 (B = −0.984 ± 0.402).

Analyses showed no significant time × group interaction for cytokines. However, multilevel analyses revealed a negative effect of IL-6 (*p* = 0.027, B = −0.285 ± 0.126) on pain intensity, detecting that an increase in IL-6 concentration results in a statistically significantly less pain intensity (timeframe T0 – T1).

A fixed-effects model showed statistically significant positive effects of lymphocytes (*p* ≤ 0.001, B = 0.676 ± 0.163) and neutrophils (*p* = 0.016, B = 0.210 ± 0.086) on IL-6 regarding the time course (T0, T1, T2).

Detailed results of blood parameter are presented in Table 2. Further analyses are presented in Tables S4–S14.

### 3.5. Physiologic Responses

A total of 18 subjects in each group, corresponding to 86% for intervention, resp. 90% for control, received the full treatment series of six interventions. Mean duration of heating-up phase in the intervention group was 45.30 ± 6.59 min (range: 35.17–57.17 min) and average plateau phase was 14.69 ± 6.64 min (range: 3.00–24.83 min). Subjects of the intervention group had a mean body-core of 38.7 ± 0.2 °C at the end of the treatment, resulting in an average rise of 1.5 ± 0.3 °C. The control group showed a highest average body-core temperature of 37.5 ± 0.3 °C, resulting in a mean rise of 0.3 ± 0.2 °C. As expected, the intervention group had a significantly higher average maximum body-core temperature (*p* < 0.001, *d* = 5.68) and rise in body-core temperature (*p* < 0.001, *d* = 5.26). There is a significant negative correlation between lymphocyte cell count (T0) and the maximum temperature reached at the first session, r = −0.510, *p* = 0.026, i.e., the lower the lymphocyte cell count (T0) was, the higher the body-core temperature was afterwards.

In addition, the intervention group showed a higher difference in baseline body-core temperature between the first and the sixth treatment, indicating a higher reduction in body-core temperature in mild WBH (*p* = 0.008, *d* = 0.98). More temperature data, pulse rate, and oxygen saturation can be found in Table S15.

### 3.6. Credibility Check

In a post hoc credibility check it appeared that all of the patients allocated to the intervention group (100%) were convinced to be part of the active condition, while 16 of 20 control patients (80%) were assuming this.

### 3.7. Safety

In the intervention group one patient missed the second treatment due to dizziness and redness after the first session of mild WBH. Another patient experienced severe dizziness and circulatory problems during the first treatment. Therefore, she received an infusion to stabilize her general condition. Side effects did not lead to a discontinuation of the treatment series in any patient of the intervention group. One patient of the control group discontinued the treatment series after the fourth session due to fatigue. A detailed presentation of side effects during the interventions, based on the reports of the therapists, is provided in Table 3. Derived and summarized data given in the pain diaries are presented in Table S16.

## 4. Discussion

### 4.1. Summary of Main Results

First, pain intensity 1 week and 6 months after the end of the treatment was significantly lower in the wIRA-WBH group compared to the control after the intervention series. A midterm sustained pain reduction can be achieved by wIRA-WBH. In addition, exploratory analyses revealed a reduction in pain impairment in the intervention group at week 30 and better mental health at week 4. Second, no severe adverse events were observed in either the intervention or the control group which indicates safety of mild wIRA-WBH, and tolerability was high. There were no drop outs due to side effects. Third, clinical results were supported by immunological data which showed a significantly higher increase in immune cell counts in the wIRA-WBH group. Higher blood lymphocyte numbers and IL-6 levels appeared with lower pain intensity.

### 4.2. Comparison to Previous Studies

The observed effects on pain intensity are in line with the previous controlled trials of wIRA-WBH in FMS [17,19,22], that showed significant group differences in pain in favor of the intervention group with moderate to large effect sizes. However, direct comparison of our findings with those from earlier studies is only possible to a limited extent due to the newly established control condition and different measurement instruments. Although the duration of the retention phase was the same, our average maximum body-core temperature at 38.7 °C was higher than Brockow’s 38.1 °C. In Romeyke’s trial, the subjects heated up even further (0.3–0.8 °C) after reaching the target temperature of 38.5 °C and maintained this temperature for about 1 h. When comparing descriptive data, the present trial showed a slightly lower reduction in pain compared to the previous works. The previous trials were conducted as part of multimodal rehabilitation programmes that consisted additional potentially pain-relieving interventions and were unblinded.

The generally mild side effects are in line with the previously reported studies on mild wIRA-WBH in FMS (e.g., Brockow et al. [17]). Body-core temperature can be increased very effectively and safely by wIRA-WBH.

The mental health summary score of SF-36 questionnaire was higher in the intervention group at week 4. This is interesting as the physical part of SF-36 showed no difference between both groups. In the previous controlled trials on WBH in FMS, Romeyke and Stummer [19] focused the most on mental effects of the therapy. They found a trend for lower depressiveness (PHQ-9) in the intervention group after an approx. two-week inpatient stay, although both groups received psychological interventions in the same amount. However, they had an average of five WBH sessions during this time. Our results on mental health are therefore partly in line with the empirical evidence. The repetition of strong stimuli is necessary for functional adaptation. The effects on mental and functional health could possibly have been stronger if patients in our trial had received the sessions with shorter intervals. However, it remains unclear why, despite pain reduction, there was no significant difference between the groups regarding physical QoL.

Though FMS is considered a chronic syndrome rather than an inflammatory disease, it exhibits distinct systemic immunological patterns, including changes in neutrophil and lymphocyte counts and elevated levels of IL-6, IL-8, and TNF [36,52]. Immune cells, such as T cells and B cells, and cytokines are clearly the key players in immune-related pain [53,54]. Cytokine levels in our trial showed no significant time × treatment interaction, although an IL-6 increase directly after intervention was detectable in the WBH group. Intervention-induced changes in lymphocyte and neutrophil counts in the WBH group may be attributed to the immunostimulatory/-modulatory effect of this therapy as described before [25]. Of note, the multilevel fixed-effects models showed statistically significant associations of lymphocytes and neutrophils with IL-6. In addition, higher concentrations of lymphocytes and IL-6 led to a lower pain intensity with evidence of statistically significant association. As Tracy et al. have shown in a meta-analysis [27], chronic pain could also be caused by parasympathetic nervous system (PNS) dysregulation, measured through the assessment of heart rate variability (HRV). A decrease in parasympathetic activation in patients with chronic pain could be detected by a decrease in HRV. These effects were particularly evident in the included studies with fibromyalgia patients. In addition, heat treatments can improve PNS function [29], which in turn plays a possible role in controlling anti-inflammatory processes [28]. It has been shown that the control of immunological processes may affect pain processing.

Thus, pain relief could have been achieved by thermotherapy-induced immune stimulation. In addition, immunological processes are supported by the higher reduction in body-core temperature in mild WBH, and may also indicate physiological adaptation as part of the strong stimulus of the therapy. Another interesting observation is the inverse correlation of lymphocyte cell number with the maximum body-core temperature reached at first session. Further studies should examine the coherence between immune system and nervous system, and the presence of chronic pain.

### 4.3. Strengths and Weaknesses

To our knowledge, the study is the first to investigate the effects of mild wIRA-WBH in patients with FMS in an outpatient setting. The main strength of the present trial was the establishment of an adequate sham condition with high credibility in the framework of a randomized controlled setting. Further, a priori sample size calculation ensured that the study was sufficiently powered to detect the group differences. The immunological data allow a combination of subjective and objective parameters and thus further supports the validity of the results.

Even if the control condition of the present trial is considered a main strength, it should be noted that it was no traditional placebo treatment, since a small amount of the postulated key factor (heat) was applied as well. However, this was considered the only way to construct a credible control condition in heat applications and effects should be limited. Besides the monocentric setting, another limitation of the present trial was that it was not fully blinded and thus a different treatment by therapists with possible influence on results cannot be excluded. However, this is not possible due to the different treatments in the two conditions and therefore only patients who had no previous hyperthermia experience were included.

## 5. Conclusions

Considering the wide range of possible applications of wIRA-WBH, the therapy is perceived to be a good treatment option to reduce pain in patients with FMS. Future research should investigate the effects of mild wIRA-WBH within a multicenter design.

## Figures and Tables

**Table 1:** Demographic and clinical characteristics.

	Mild WBH (*n* = 21)	Sham Hyperthermia (*n* = 20)
Sex (female/male)	19/2	20/0
Age, *M* (*SD*), years	54.61 (7.65)	56.40 (4.86)
BMI, *M* (*SD*), kg/m^2^	28.61 (6.70)	27.47 (7.05)
Education, *n* (%)		
Primary school graduation	9 (42.9)	3 (15)
Secondary school certificate	7 (33.3)	14 (70)
Qualification for university entrance	3 (9.5)	3 (15)
University degree	2 (9.5)	0 (0)
Marital status, *n* (%)		
Single	2 (9.5)	3 (15)
Married/partner	15 (71.4)	14 (70)
Divorced, separated, widowed	4 (19)	3 (15)
Employment status, *n* (%)		
Employed	15 (71.4)	14 (70)
Employed, but sick leave	1 (4.8)	3 (15)
Unemployed	0 (0)	0 (0)
Retired, housework, apprentice	6 (28.6)	6 (30)
Comorbidities, *n* (%)		
Metabolic	7 (33.3)	8 (40)
Psychiatric	8 (38.1)	5 (25)
Nervous system	13 (61.9)	9 (45)
Cardiovascular	5 (23.8)	3 (15)
Gastrointestinal	5 (23.8)	1 (5)
Musculoskeletal	12 (57.1)	10 (50)
Other	7 (33.3)	7 (35)
Medication, *n* (%)		
Opioid Analgesics	3 (14.3)	0 (0)
Non-opioid Analgesics	8 (38.1)	7 (35)
NSAID	11 (52.4)	14 (70)
Muscle relaxants	0 (0)	0 (0)
Anticonvulsants	3 (14.3)	0 (0)
Antidepressants	8 (38.1)	7 (35)
Glucocorticoids	0 (0)	1 (5)
Other	15 (71.4)	12 (60)
Pain intensity (1–10), *M* (*SD*)	5.53 (1.40)	5.26 (0.95)

Note. *n* = sample size, *M* = mean, *SD* = standard deviation, BMI = body mass index.

**Table 2:** Circulating cytokines, immune cells, and inflammatory markers.

Outcome	Group	T0/Baseline (Week 0)	T1 (End of Treatment)	T2 (Week 4)
TNF	Hyperthermia	0.88 ± 0.25	0.93 ± 0.24	0.99 ± 0.32
	Sham Hyperthermia	0.84 ± 0.16	0.88 ± 0.25	0.95 ± 0.26
IL-6	Hyperthermia	2.00 ± 1.35	3.04 ± 1.91	2.31 ± 1.45
	Sham Hyperthermia	2.01 ± 1.68	2.52 ± 2.20	2.18 ± 1.67
IL-8	Hyperthermia	6.56 ± 2.92	7.35 ± 3.55	6.32 ± 1.56
	Sham Hyperthermia	6.24 ± 1.88	6.90 ± 2.03	6.47 ± 1.65
IL-10	Hyperthermia	1.52 ± 1.64	1.56 ± 1.81	2.24 ± 2.03
	Sham Hyperthermia	1.01 ± 1.15	1.15 ± 1.07	1.79 ± 3.42
Neutrophils	Hyperthermia	4.47 ± 1.92	4.50 ± 1.95	4.82 ± 2.77
	Sham Hyperthermia	3.88 ± 0.95	3.79 ± 0.76	3.38 ± 0.86
Leukocytes	Hyperthermia	7.33 ± 2.25	8.60 ± 2.95	8.04 ± 3.23
	Sham Hyperthermia	6.31 ± 1.21	6.75 ± 1.36	6.26 ± 1.08
Lymphocytes	Hyperthermia	2.08 ± 0.49	2.91 ± 0.71	2.34 ± 0.54
	Sham Hyperthermia	1.85 ± 0.32	2.20 ± 0.62	2.06 ± 0.35
Monocytes	Hyperthermia	0.54 ± 0.15	0.53 ± 0.36	0.57 ± 0.38
	Sham Hyperthermia	0.48 ± 0.20	0.51 ± 0.26	0.51 ± 0.21
Thrombocytes	Hyperthermia	282.95 ± 49.14	294.99 ± 53.78	282.42 ± 41.28
	Sham Hyperthermia	263.60 ± 43.64	256.01 ± 34.32	263.81 ± 36.83
CRP	Hyperthermia	0.31 ± 0.39	0.22 ± 0.33	0.23 ± 0.39
	Sham Hyperthermia	0.29 ± 0.28	0.20 ± 0.23	0.18 ± 0.20
ESR	Hyperthermia	9.95 ± 6.52	8.68 ± 4.59	8.97 ± 4.85
	Sham Hyperthermia	10.20 ± 6.14	11.43 ± 7.68	10.57 ± 4.51

Note: The table displays the means and standard deviations (m ± sd) of the cytokine (plasma), immune cell and inflammatory marker outcomes from venous blood sample over the three measurement times. Cytokines are presented as pg/mL; immune cell populations are presented as cells x 10^3^/µL; CRP is presented as mg/dL, ESR is presented as mm/1 h. Sample size *n* = 41 (Hyperthermia *n* = 21, sham hyperthermia *n* = 20).

**Table 3:** Treatment intensity, duration, and side effects during interventions.

	Mild WBH *M* ± *SD*	Sham Hyperthermia *M* ± *SD*
Heating phase (min) ^a^	45.30 ± 6.69	-
Retention phase (min) ^a^	14.69 ± 6.64	-
Maximum Temperature (°C) ^a^	38.74 ± 0.17	37.52 ± 0.25
Side effects during interventions, *n* (%) ^b^		
Burning sensation on skin (calf, buttocks, shoulder, back)	15 (71.4)	0
Physical stress (feeling of heat/palpitations/throbbing/restlessness)	8 (38.1)	0
Headache	5 (23.8)	4 (20)
Severe circulatory problems	2 (9.5)	0
Dizziness	2 (9.5)	0
Poor general condition	1 (4.8)	0
Tingling/shaking	1 (4.8)	0

Note. *M* = mean, *SD* = standard deviation; ^a^ sample size *n* = 40 (mild WBH *n* = 20, sham hyperthermia *n* = 20); ^b^ multiple mentions per person possible.

## Data Availability

Data will be provided by the corresponding author on reasonable request.

## Supplementary Materials

The following are available online at https://www.mdpi.com/article/10.3390/jcm12082945/s1. Figure S1. Flow chart. Table S1. Inclusion and exclusion criteria. Table S2. Description of the deployed questionnaires. Table S3. Secondary outcomes. Table S4. Blood analyses of immune cells and inflammatory markers_rmANOVA multivariate. Table S5. Blood analyses of immune cells and inflammatory markers_rmANOVA univariate. Table S6. Post hoc *t*-tests for lymphocytes. Table S7. Post hoc T-tests for neutrophils. Table S8. Blood analyses of cytokines_rmANOVA multivariate. Table S9. Fixed-effects model of lymphocytes and neutrophils on pain intensity_T0T1. Table S10. Fixed-effects model of lymphocytes and neutrophils on pain intensity_T0T2. Table S11. Fixed-effects model of cytokines on pain intensity_T0T1. Table S12. Fixed-effects model of cytokines on pain intensity_T0T2. Table S13. Fixed-effects model of neutrophils and lymphocytes on interleukin-6. Table S14. Circulating cytokines, immune cells, and inflammatory markers. Table S15. Physiological responses. Table S16. Side effects within 24 h of interventions.
