# Supplementary material for: Mild Water-Filtered Infrared-A Whole-Body Hyperthermia Reduces Pain in Patients with Fibromyalgia Syndrome—A Randomized Sham-Controlled Trial
Source: J Clin Med. 2023 Apr 18;12(8):2945. doi: 10.3390/jcm12082945 (PMC10144038; doi:10.3390/jcm12082945)
Supplement: Supplementary file 1 [file jcm-12-02945-s001.zip › jcm-2282176-supplementary.pdf]

Supplementary Materials

Table S1. Inclusion and exclusion criteria.

Inclusion criteria	Exclusion Criteria
- Age between 18 – 70 years<br>- Confirmed diagnosis of fibromyalgia syndrome<br>- Average pain intensity ≥ 4.0<br>- Signed declaration of consent	- Participation in other clinical studies<br>- Previous experience with whole-body hyperthermia<br>- Contradictions for hyperthermia (severe cardiovascular diseases, cancer, acute infections, pregnant and breastfeeding woman)<br>- Acute and/ or feverish microbial infections<br>- Patients with severe, rheumatic, endocrine, neurological (especially if associated with cognitive disorders), liver, kidney and cardiac concomitant diseases<br>- Patients who are permanently treated with opioids, cannabis, immunosuppressive drugs, Alpha/Beta-A(nta)gonists due to a disease from the group described above<br>- Pain due to severe psychiatric (bipolar disorder, psychosis, personality disorder, severe depression, substance abuse), systemic and neurological disorders

Table S2. Description of the deployed questionnaires

Questionnaire	Outcome	Description
Brief Pain Inventory (BPI)	Pain Intensity	The subscale describes a mean score ranging from 1-10 covering the "strongest", "lowest" and "average" pain of the past 24 hours and the current pain. Each of the four items uses a numerical analogue scale (NRS) from 1 (*no pain*) to 10 (*worst pain imaginable*). Higher scores indicate higher average pain intensity.
Brief Pain Inventory (BPI)	Pain Impairment	It refers to an average score based on seven NRS-items from 1 (*does not interfere*) – 10 (*completely interferes*). It covers pain impairment in the areas general activity, mood, walking ability, work,

		relationships, sleep and enjoyment of life. Higher mean scores indicate higher interference.
Fibromyalgia Impact Questionnaire (FIQ)	Fibromyalgia-related Quality of Life	The FIQ used in this study contains eight subscales referring to the past week: physical impairment (10 Sub-Items), feel good, pain, fatigue, morning tiredness, stiffness, anxiety and depression. An 8-subscales adapted sum score can be calculated from 0–100 without the two work items with higher scores representing a higher impact of FMS on quality of life. We multiplied the total FIQ score by 1.25 so that results can be compared across studies.
Patient Health Questionnaire (PHQ-9)	Depression	It contains nine items that correspond to the DSM-4 criteria for depression. Each item is rated by the respondent from 0 (*not at all*) to 3 (*almost every day*). The total score of the questionnaire ranges from 0 to 27 and can be classified as mild depression at a score of ≥ 5, moderate depression at ≥ 10, moderately severe depression at ≥ 15, and severe depression at ≥ 20.
Multidimensional Fatigue Inventory (MFI-20)	Fatigue	The questionnaire contains five subscales: general fatigue, physical fatigue, reduced motivation, reduced activity and mental fatigue, which are assessed on 5-point Likert-Scales. Subscales range from 4-20 points, which can be summarized in a total score ranging from 20-100 with higher scores indicating higher fatigue.
Pittsburgh Sleep Quality Index (PSQI)	Sleep Quality	It contains seven subscales: subjective sleep quality, sleep latency, sleep duration, habitual sleep efficiency, sleep disturbances, use of sleeping medication, and daytime dysfunction. All items refer to the last four weeks and are assessed either in four frequency levels or in an open-end format. A total sum score from 0 – 21 can be derived from the subscales with higher scores indicating higher sleep disturbance.
Short Form Health Survey (SF-36)	General health-related Quality of Life	The SF-36 contains eight subscores: physical functioning (10 items), bodily pain (two items), general health perception (five items), physical role limitations (four items), emotional role limitations (three items), social functioning (two items), vitality (four items) and mental health (five items), which are assessed either on 5-point Likert scales, 3-point Likert scales or with dichotomous items. Higher subscales indicate better health status. In addition, two component summaries (physical and mental) can be calculated using weighted subscales. The eight domains were scored on a 0–100 scale while the two summary measures are norm-based T-scores, with a mean of 50 and standard deviation of 10. In all cases, higher scores indicated better health-related quality of life.
Generalized Anxiety Disorder Scale (GAD-7)	Anxiety	With seven items it covers the main DSM-4 criteria of a generalized anxiety disorder. Each item is rated by the respondent from 0 (*not at all*) to 3 (*almost every day*). A total sum score can be derived ranging from 0 – 21, with 5 – 9 representing mild, 10 – 14 moderate, and >= 15 points severe symptoms.
Short-Form McGill Pain Questionnaire (SF-MPQ)	Sensory and Affective Pain	It contains 15 items, while 11 items cover sensory and 4 items comprise affective pain. Two subscores can be derived ranging from 0 – 33 (sensory) and 0 – 12 (affective) with higher scores indicating higher pain.

Patient Health Questionnaire-15 (PHQ-15)	Somatic Symptom Load	It contains 13 items covering various physical complaints and two items of the depression module (PHQ-9). Items are answered by the respondents from 0 (*not bothered at all*) to 2 (*bothered a lot*). A sum score can be calculated from 0 – 30 with higher scores representing higher somatic symptom load.

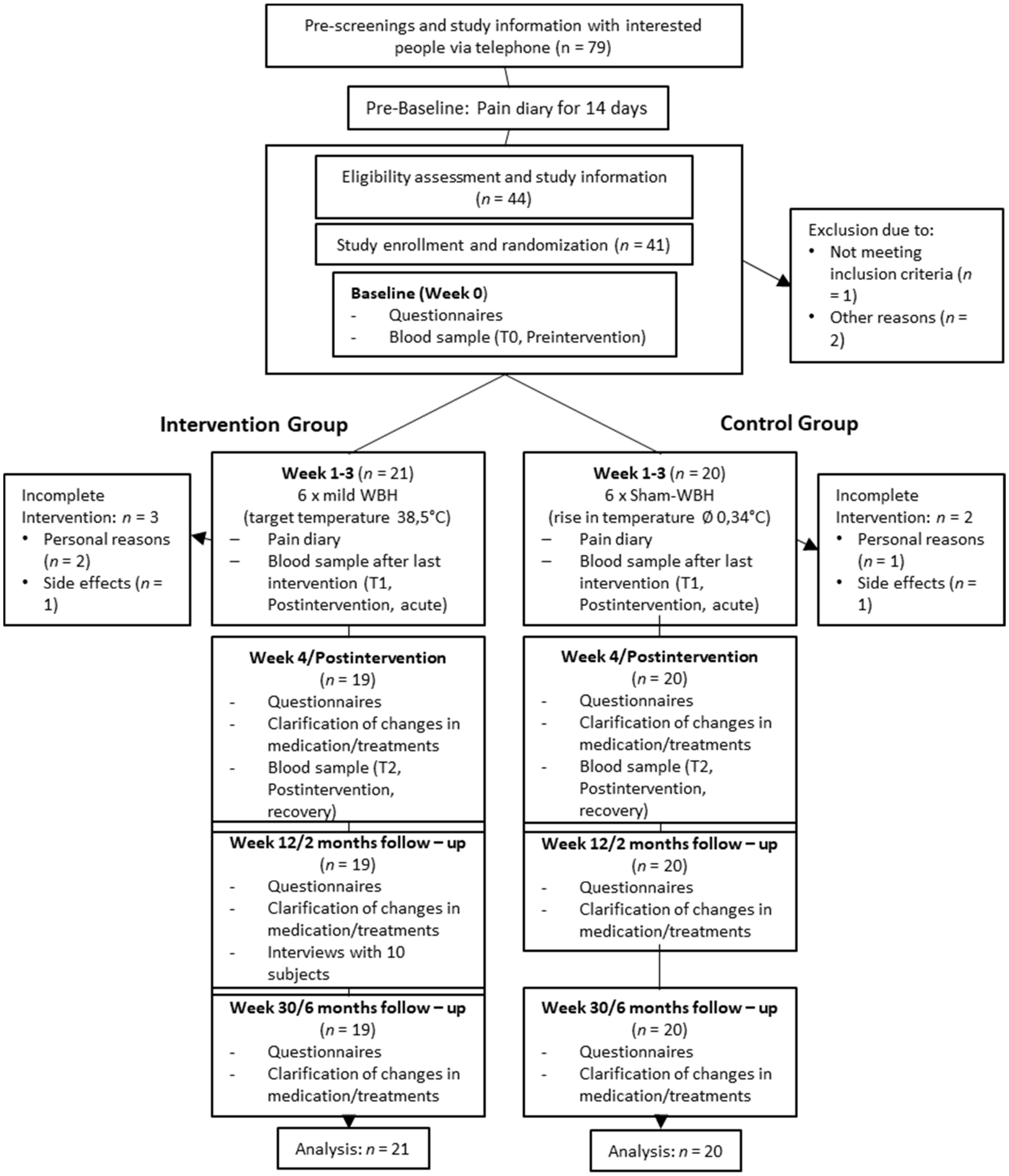

Figure S1. Flow chart .

Table S3. Secondary outcomes

	Baseline (Week 0)	Post (Week 4)	Follow-Up (Week 12)	Follow-Up (Week 30)	Group differences week 4		Group differences week 12		Group differences week 30	
					p	η^2_p	*p*	η^2_p	*p*	η^2_p
**Pain Intensity** (BPI)										
Hyperthermia	5.53±1.40	3.83±1.64	4.41±1.83	4.59±1.80	.015*	.146	.096	.071	.002**	.233
Sham-Hyperthermia	5.26±0.95	4.76±1.92	4.93±1.75	5.80±1.65						
**Pain Impairment** (BPI)										
Hyperthermia	5.27±1.91	3.37±2.23	4.27±2.45	3.96±2.45	.125	.061	.277	.031	.008**	.172
Sham-Hyperthermia	4.32±1.28	3.77±1.86	4.48±1.43	5.13±1.77						
**Sensory/Affective Pain** (SF-MPQ)										
Hyperthermia	32.06±7.63	20.24±8.05	23.34±9.11	25.11±12.00	.147	.054	.070	.084	.115	.064
Sham-Hyperthermia	31.28±8.30	23.17±11.51	27.31±12.02	30.17±11.21						
**FM-related QoL** (FIQ)										
Hyperthermia	63.65±11.85	37.49±16.47	45.13±17.03	48.27±20.28	.245	.035	.060	.090	.135	.058
Sham-Hyperthermia	56.10±14.37	42.00±20.47	49.98±16.13	53.86±18.59						
**HLQOL Physical** (SF-36)										
Hyperthermia	29.07±6.15	33.24±8.92	33.86±9.47	34.22±9.65	.547	.010	.340	.025	.200	.043
Sham-Hyperthermia	30.53±6.63	33.55±8.28	33.06±7.44	32.68±8.92						
**HLQOL Psychological** (SF-36)										
Hyperthermia	38.77±11.60	49.50±9.47	44.09±12.26	44.80±12.77	.043*	.103	.533	.011	.222	.039
Sham-Hyperthermia	43.88±9.72	47.12±12.35	44.59±9.95	42.50±11.48						
Physical Functioning Index										
Hyperthermia	45.95±19.01	55.62±18.54	59.14±19.63	57.90±22.25	.348	.023	.230	.038	.437	.016
Sham-Hyperthermia	50.50±20.45	54.25±22.02	54.50±19.26	55.00±21.09						
Role-Physical Index										
Hyperthermia	9.52±16.73	36.10±33.90	33.95±32.85	32.95±40.90	.751	.003	.134	.058	.386	.020
Sham-Hyperthermia	15.00±20.52	37.50±41.75	25.00±30.35	27.50±37.08						
Bodily Pain Index										
Hyperthermia	25.81±9.72	44.53±17.86	38.84±18.02	42.67±17.71	.223	.041	.433	.016	.008**	.181
Sham-Hyperthermia	30.45±12.41	39.95±18.79	38.95±17.57	29.55±16.94						
General Perception Index										
Hyperthermia	36.52±12.72	44.10±18.30	42.62±17.79	41.69±17.54	.145	.055	.418	.017	.585	.008
Sham-Hyperthermia	46.00±17.71	44.90±15.70	47.20±20.05	45.50±19.05						
Vitality Index										
Hyperthermia	23.57±14.93	41.19±19.74	31.03±18.44	33.62±20.84	.339	.024	.765	.002	.407	.018
Sham-Hyperthermia	31.42±14.50	38.08±16.13	33.25±18.87	31.33±19.59						
Social Functioning Index										
Hyperthermia	47.83±23.99	68.10±19.79	63.31±28.56	65.48±26.19	.224	.039	.337	.024	.048*	.099
Sham-Hyperthermia	56.88±22.02	68.13±26.12	60.00±28.56	56.25±27.66						

Role-Emotional Index										
Hyperthermia	41.27±47.03	79.10±30.33	60.32±38.54	64.27±42.90	.056	.093	.371	.021	.283	.030
Sham-Hyperthermia	53.33±41.04	63.33±45.76	56.67±44.72	51.67±45.21						
Mental Health Index										
Hyperthermia	51.62±16.24	66.90±15.60	63.67±18.93	61.05±20.76	.102	.069	.400	.019	.282	.030
Sham-Hyperthermia	60.80±17.20	66.00±18.69	63.80±14.36	60.00±18.67						
**Fatigue (MFI-20)**										
Hyperthermia	73.95±11.31	66.00±14.63	65.86±14.01	67.14±14.96	.426	.017	.202	.042	.270	.032
Sham-Hyperthermia	65.80±10.19	63.85±13.74	66.75±12.08	68.10±14.46						
General Fatigue (MFI-20)										
Hyperthermia	16.14±2.57	14.43±3.53	14.95±3.69	14.95±3.64	.432	.016	.299	.028	.256	.034
Sham-Hyperthermia	15.05±3.02	14.80±3.81	15.55±3.97	15.65±3.36						
Physical Fatigue (MFI-20)										
Hyperthermia	16.14±2.76	14.29±3.52	13.95±3.29	14.38±3.84	.506	.012	**.017***	.141	.140	.056
Sham-Hyperthermia	14.90±2.85	14.15±3.48	14.90±3.52	15.20±4.15						
Motivation (MFI-20)										
Hyperthermia	14.62±3.12	14±3.27	13.71±2.76	13.48±3.19	.744	.003	.915	.000	.119	.063
Sham-Hyperthermia	13.55±2.95	13.05±3.55	13.50±3.05	14.45±4.22						
Activity (MFI-20)										
Hyperthermia	12.14±3.51	10.33±3.21	10.62±2.97	10.57±3.87	.639	.006	.507	.012	.421	.017
Sham-Hyperthermia	10.20±3.29	9.55±3.32	10.40±3.22	10.80±3.40						
Mental Fatigue (MFI-20)										
Hyperthermia	14.90±3.35	12.95±4.26	12.62±4.19	13.76±3.78	.342	.024	.251	.035	.924	.000
Sham-Hyperthermia	12.10±3.78	12.30±3.94	12.40±3.90	12.00±3.97						
**Sleep** Quality (PSQI)										
Hyperthermia	11.90±3.43	9.29±4.55	9.95±4.28	10.81±3.71	.063	.088	.148	.054	.192	.044
Sham-Hyperthermia	10.55±4.27	9.55±4.97	10.40±3.79	11.15±3.73						
Depression (PHQ-9)										
Hyperthermia	13.38±4.27	7.67±4.55	9.24±5.47	4.52±4.50	.075	.081	.131	.059	.399	.019
Sham-Hyperthermia	11.00±4.00	9.05±4.65	9.75±3.18	4.55±2.72						
**Somatization** (PHQ-15)										
Hyperthermia	16.43±4.74	12.81±4.92	13.71±5.40	14.10±5.23	.286	.030	.200	.043	.117	.063
Sham-Hyperthermia	14.40±3.73	12.65±3.69	13.60±3.07	13.80±3.09						
**Anxiety (GAD-7)**[a]										
Hyperthermia	9.67±4.17	5.60±4.82	7.13±4.73	7.33±5.37	.177	.072	.137	.086	.228	.058
Sham-Hyperthermia	7.00±4.26	5.15±3.41	7.08±3.88	7.23±4.40						

Note: N = sample size; questionnaires filled out by N = 41 (Hyperthermia: N = 21; Sham-Hyperthermia: N = 20); [a]GAD-7 questionnaire filled out by N = 28 (Hyperthermia: N = 15; Sham-Hyperthermia: N = 13); all results are reported as mean ± standard deviation; significant results are in **bold** (significance * ≤ .05, ** ≤ .01, *** ≤ .001)

Table S4. Blood analyses of immune cells and inflammatory markers_rmANOVA multivariate.

Predictor	Value	F	Hypothesis df	Error df	Sig.	Partial Eta-squared
Measurement time	.581	4.27	14.00	146.00	**<.001***	.291

Measurement time * group	.344	2.17	14.00	146.00	**.012***	.172

Note: The table displays the results of the repeated measures ANOVA (rmANOVA) over the three measurement times. Dependent variables were CRP, ESR, Lymphocytes, Leukocytes, Monocytes, Thrombocytes and Neutrophils (***significance ≤.001, * significance ≤.05). Sample size N = 41.

Table S5. Blood analyses of immune cells and inflammatory markers_rmANOVA univariate.

Source of variance	Measure	Type III Sum of Squares	df	Mean square	F	Sig.	Partial Eta-Square
Measurement time	CRP	.234	2	.117	3.69	**.029***	.086
	ESR	2.37	2	1.19	.155	.856	.004
	Lymphocytes	7.20	2	3.60	25.12	**<.001*****	.392
	Leukocytes	15.20	2	7.60	6.75	**.002****	.148
	Monocytes	.020	2	.010	.336	.715	.009
	Thrombocytes	145.74	2	72.87	.122	.886	.003
	Neutrophils	.118	2	.059	.088	.916	.002
Measurement time * group	CRP	.008	2	.004	.119	.888	.003
	ESR	32.13	2	16.06	2.10	.129	.051
	Lymphocytes	1.45	2	.725	5.06	**.009****	.115
	Leukocytes	4.37	2	2.19	1.94	.151	.047
	Monocytes	.012	2	.006	.203	.817	.005
	Thrombocytes	2734.75	2	1367.37	2.28	.109	.055
	Neutrophils	4.33	2	2.16	3.23	**.045***	.077

Note: The table displays the results of the subsequent univariate repeated measures ANOVA (rmANOVA) over the three measurement times. Significant results are in **bold** (*** significance ≤.001, ** significance ≤.01 *significance ≤.05). Sample size N = 41.

Table S6. Post-hoc T-tests for Lymphocytes.

				95% Confidence Interval	
	T	df	Sig. (2-sided)	Lower bound	Upper bound
Lymphocytes, T0	- 1.78	39	.082	-.500	.031
Lymphocytes, T1	- 3.45	39	**.001*****	-1.14	-.296
Lymphocytes, T2	- 1.98	39	.055	-.571	.006

Note: The table displays the results of the Post-hoc T-Tests for group differences at three measurement timepoints. Significant results are in **bold** (*** significance ≤.001). Sample size N = 41.

Table S7. Post-hoc T-tests for Neutrophils.

				95% Confidence Interval	
	T	df	Sig. (2-sided)	Lower bound	Upper bound
Neutrophils, T0	- 1.23	39	.225	-1.56	.377
Neutrophils, T1	- 1.54	26.26	.135	-1.65	.235
Neutrophils, T2	- 2.27	24.04	**.033***	-2.75	-.130

Note: The table displays the results of the Post-hoc T-Tests for group differences at three measurement timepoints. Significant results are in **bold** (*significance ≤.05). Sample size N = 41.

Table S8. Blood analyses of cytokines_rmANOVA multivariate.

Predictor	Value	F	Hypothesis df	Error df	Sig.	Partial Eta-squared
Measurement time	.379	3.972	8.000	136.000	**<.001*** **	.189
Measurement time*group	.061	.533	8.000	136.000	.830	.030

Note: The table displays the results of the repeated measures ANOVA (rmANOVA) over the three measurement times. Dependent variables were TNF, IL-6, IL-8 and IL-10. Significant results are in **bold** (***significance ≤.001). Sample size N = 41.

Table S9. Fixed-effects model of lymphocytes and neutrophils on pain intensity_T0T1.

						95% Confidence Interval	
Predictor	Value	F	Hypothesis df	Error df	Sig.	Lower Bound	Upper Bound
Constant	6.30	.714	71.70	8.83	.000	4.88	7.72
Lymphocytes	-.905	.257	65.36	-3.52	**.001*** **	-1.42	-.391
Neutrophils	.141	.142	65.77	.997	.322	-.142	.424
	AIC	Layers					
Full model	306.02	5					

Note: For the fixed-effects model values from T0 and T1 were applied for lymphocytes and neutrophils. The dependent variable was the BPI summary score (pain intensity); AIC = Akaike's information criterion; significant results are in **bold** (***significance ≤ .001). Sample size N = 41.

Table S10. Fixed-effects model of lymphocytes and neutrophils on pain intensity_T0T2.

						95% Confidence Interval	
Predictor	Value	F	Hypothesis df	Error df	Sig.	Lower Bound	Upper Bound
Constant	6.75	.911	74.83	7.41	.000	4.93	8.56
Lymphocytes	-.984	.402	78.95	-2.45	**.017***	-1.78	-.184
Neutrophils	.035	.111	62.86	.313	.755	-.187	.256
	AIC	Layers					
Full model	310.70	5					

Note: For the fixed-effects model values from T0 and T2 were applied for lymphocytes and neutrophils. The dependent variable was the BPI summary score (pain intensity); AIC = Akaike's information criterion; significant results are in **bold** (*significance ≤ .05). Sample size N = 41.

Table S11. Fixed-effects model of cytokines on pain intensity_T0T1.

						95% Confidence Interval	
Predictor	Value	F	Hypothesis df	Error df	Sig.	Lower Bound	Upper Bound
Constant	5.42	.892	53.85	6.08	.000	3.63	7.21
TNF	.199	1.06	64.21	.188	.851	-1.91	2.31
IL-6	-.285	.125	61.00	-2.27	**.027***	-.537	-.034
IL-8	.000	.085	56.32	.002	.998	-.170	.170
IL-10	-.056	.141	52.39	-.399	.692	-.340	.227
	AIC	Layers					

Full model	312.82	7

Note: For the fixed-effects model values from T0 and T1 were applied for cytokines. The dependent variable was the BPI summary score (pain intensity); AIC = Akaike's information criterion; significant results are in **bold** (*significance ≤ .05). Sample size N = 41.

Table S12. Fixed-effects model of cytokines on pain intensity_T0T2.

						95% Confidence Interval	
**Predictor**	**Value**	**F**	**Hypothesis df**	**Error df**	**Sig.**	**Lower Bound**	**Upper Bound**
Constant	5.76	.842	58.74	6.84	.000	4.08	7.45
TNF	-1.24	.920	68.01	-1.35	.183	-3.07	.597
IL-6	-.097	.154	51.76	-.633	.530	-.406	.211
IL-8	.092	.100	68.13	.911	.365	-.109	.292
IL-10	-.104	.085	76.19	-1.22	.225	-.275	.066
	AIC	Layers					
Full model	313.06	7					

Note: For the fixed-effects model values from T0 and T2 were applied for cytokines. The dependent variable was the BPI summary score (pain intensity); AIC = Akaike's information criterion. Sample size N = 41.

Table S13. Fixed-effects model of Neutrophils and Lymphocytes on Interleukin-6.

						95% Confidence Interval	
**Predictor**	**Value**	**F**	**Hypothesis df**	**Error df**	**Sig.**	**Lower Bound**	**Upper Bound**
Constant	-.118	.518	118.70	-.227	.821	-1.14	.909
Neutrophils	.210	.086	117.76	2.45	**.016***	.040	.379
Lymphocytes	.676	.163	86.52	4.15	**<.001*****	.352	1.00
	AIC	Layers					
Full model	405.46	6					

Note: For the fixed-effects model values from three timepoints (T0, T1, T2) were applied for Neutrophils, Lymphocytes and Interleukin-6 (IL-6). The dependent variable was IL-6; AIC = Akaike's information criterion; significant values are in **bold** (***significance ≤.001, *significance ≤ .05). Sample size N = 41.

Table S14. Circulating cytokines, immune cells and inflammatory markers.

**Outcome**	**Group**	**T0/Baseline (week 0)**	**T1 (end of treatment)**	**T2 (week 4)**
**TNF**	Hyperthermia	0.88 ± 0.25	0.93 ± 0.24	0.99 ± 0.32
	Sham-Hyperthermia	0.84 ± 0.16	0.88 ± 0.25	0.95 ± 0.26
**IL-6**	Hyperthermia	2.00 ± 1.35	3.04 ± 1.91	2.31 ± 1.45
	Sham-Hyperthermia	2.01 ± 1.68	2.52 ± 2.20	2.18 ± 1.67
**IL-8**	Hyperthermia	6.56 ± 2.92	7.35 ± 3.55	6.32 ± 1.56
	Sham-Hyperthermia	6.24 ± 1.88	6.90 ± 2.03	6.47 ± 1.65
**IL-10**	Hyperthermia	1.52 ± 1.64	1.56 ± 1.81	2.24 ± 2.03
	Sham-Hyperthermia	1.01 ± 1.15	1.15 ± 1.07	1.79 ± 3.42
**Neutrophils**	Hyperthermia	4.47 ± 1.92	4.50 ± 1.95	4.82 ± 2.77
	Sham-Hyperthermia	3.88 ± 0.95	3.79 ± 0.76	3.38 ± 0.86
**Leukocytes**	Hyperthermia	7.33 ± 2.25	8.60 ± 2.95	8.04 ± 3.23

	Sham-Hyperthermia	6.31 ± 1.21	6.75 ± 1.36	6.26 ± 1.08
**Lymphocytes**	Hyperthermia	2.08 ± 0.49	2.91 ± 0.71	2.34 ± 0.54
	Sham-Hyperthermia	1.85 ± 0.32	2.20 ± 0.62	2.06 ± 0.35
**Monocytes**	Hyperthermia	0.54 ± 0.15	0.53 ± 0.36	0.57 ± 0.38
	Sham-Hyperthermia	0.48 ± 0.20	0.51 ± 0.26	0.51 ± 0.21
**Thrombocytes**	Hyperthermia	282.95 ± 49.14	294.99 ± 53.78	282.42 ± 41.28
	Sham-Hyperthermia	263.60 ± 43.64	256.01 ± 34.32	263.81 ± 36.83
**CRP**	Hyperthermia	0.31 ± 0.39	0.22 ± 0.33	0.23 ± 0.39
	Sham-Hyperthermia	0.29 ± 0.28	0.20 ± 0.23	0.18 ± 0.20
**ESR**	Hyperthermia	9.95 ± 6.52	8.68 ± 4.59	8.97 ± 4.85
	Sham-Hyperthermia	10.20 ± 6.14	11.43 ± 7.68	10.57 ± 4.51

Note: The table displays the means and standard deviations (m ± sd) of the cytokine (plasma), immune cell and inflammatory marker outcomes from venous blood sample over the three measurement times. Cytokines are presented as pg/ml; Immune cell populations are presented as cells $\chi 10^3$/µl; CRP is presented as mg/dl, ESR is presented as mm/1h. Sample size N = 41 (Hyperthermia N = 21, Sham-Hyperthermia N = 20).

Table S15. Physiological responses.

	Mild WBH	Sham Hyperthermia	t-Test p – value (two-sided)	95% Confidence Interval		Cohen's d
	M ± SD	M ± SD		Lower Bound	Upper Bound	
Heating phase (min)	45.30 ± 6.69	-	-	-	-	-
Retention phase (min)	14.69 ± 6.64	-	-	-	-	-
Base Temperature (°C)	37.17 ± 0.20	37.18 ± 0.33	.953	-.18	.17	- .019
Base Pulse (bpm)	76.75 ± 6.26	75.22 ± 10.67	.584	-4.11	7.17	.175
Base SpO2 (%)	98.80 ± 0.73	98.76 ± 0.90	.868	-.48	.57	.053
Maximum Temperature (°C)	38.74 ± 0.17	37.52 ± 0.25	<.001	1.08	1.36	5.675
Maximum Pulse (bpm)	114.17 ± 12.53	90.33 ± 9.49	<.001	16.72	30.96	2.145
Maximum SpO2 (%)	99.65 ± 0.35	99.65 ± 0.33	.964	-.22	.23	.015
T30 (°C)	38.35 ± 0.18	37.37 ± 0.27	<.001	.83	1.12	4.216
End pulse (bpm)	101.14 ± 10.42	81.60 ± 8.59	<.001	13.44	25.66	2.048
End SpO2 (%)	98.25 ± 0.95	97.85 ± 1.09	.222	-.25	1.06	.393
Δ base temp 1th – 6th treatment (°C)	0.27 ± 0.25	0.03 ± 0.26	.008	.07	.43	.980

Note: M = mean, SD = standard deviation, T30 = temperature over 30 minutes, [a]Sample size N = 40 (Mild WBH N = 20, Sham-Hyperthermia N = 20), [b]Sample size, Mild WBH N = 18, Sham Hyperthermia N = 16.

Table S16. Side effects within 24 hours after interventions.

	Mild WBH	Sham-WBH
	N (%)	*N* (%)
Fatigue	7 (33,3)	3 (15)
Sweating	2 (9,5)	0
Dizziness	2 (9,5)	0
Headache	2 (9,5)	0
Pain	2 (9,5)	0
Nausea	1 (4,8)	0
Burning Sensations/ Redness	1 (4,8)	0
Circulatory problems	1 (4,8)	1 (5)

Sore Muscles	1 (4,8)	1 (5)
Tingling	1 (4,8)	1 (5)
Photosensibility	1 (4,8)	0 (5)

Note: multiple mentions per person possible, N = number.
